# Naturally occurring parasitoids of *Drosophila suzukii* (Diptera: Drosophilidae) and other drosophilids in California fruit regions

**DOI:** 10.1093/jee/toaf132

**Published:** 2025-06-20

**Authors:** Kent M Daane, Brian N Hogg, Judith M Stahl, David R Haviland, Xingeng Wang

**Affiliations:** Department of Environmental Science, Policy and Management, University of California, Berkeley, CA, USA; USDA Agricultural Research Service, Invasive Species and Pollinator Health Research Unit, Albany, CA, USA; Health & Biosecurity, CSIRO, Canberra, Australian Capital Territory, Australia; University of California Cooperative Extension, Kern County, Bakersfield, CA, USA; USDA Agricultural Research Service, Beneficial Insects Introduction Research, Newark, DE, USA

**Keywords:** biological control, invasive species, parasitoids, spotted-wing drosophila

## Abstract

*Drosophila suzukii* (Matsumura) (Diptera: Drosophilidae) has become a damaging economic pest of small fruits in the invaded range in the Americas, Europe, and North Africa. This study surveyed naturally occurring parasitoids of *D. suzukii* and other frugivorous Drosophilidae in California’s coastal and interior fruit production regions. Surveys were conducted from 2012 to 2018 through collections of infested fruits and the use of sentinel fruit or host traps. Two pupal parasitoids, *Pachycrepoideus vindemiae* (Rondani) (Hymenoptera: Pteromalidae) and *Trichopria drosophilae* (Perkins) (Hymenoptera: Diapriidae) were collected from *D. suzukii* and other drosophilids; the former species dominated in the interior while the latter species dominated in the coastal region. Two larval parasitoids, *Leptopilina boulardi* Barbotin et al. and *L. heterotoma* (Thomson) (Hymenoptera: Figitidae) were collected from drosophilid species other than *D. suzukii*, and were the dominant parasitoids in the interior and coastal regions, respectively. These four common parasitoids were most active during the spring and fall. The levels of parasitism on *D. suzukii* were < 10% from field-collected fruits but were as high as 74% in sentinel traps. Pupal parasitoids attacked *D. suzukii* at a higher rate than *D. melanogaster* in sentinel traps baited with both fly species. These results are discussed in conjunction with ongoing efforts to introduce larval parasitoids from the fly’s native range.

## Introduction

Native to East Asia, the invasive *Drosophila suzukii* (Matsumura) (Diptera: Drosophilidae), has been widely established in the Americas, Europe, and North Africa (Asplen et al. 2015, Boughdad et al. 2021, Garcia et al. 2022). It feeds on essentially all soft-skinned fruit, including blackberries, blueberries, cherries, raspberries, strawberries, and various wild host plants (Stewart et al. 2014, Lee et al. 2015, Poyet et al. 2015, Kenis et al. 2016). Its serrated ovipositor allows it to oviposit in ripe fruit, unlike other drosophilid flies, such as *D. melanogaster* Meigen (Diptera: Drosophilidae), that only attack damaged and/or rotting fruit (Atallah et al. 2014). The rapid development and high reproductive potential of *D. suzukii* can lead to explosive population increases and significant economic loss to commercial crops (Tait et al. 2021).

Alternative control approaches, including behavioral and cultural methods, have been implemented to suppress *D. suzukii* populations and reduce crop damage (reviewed by Haye et al. 2016, Tait et al. 2021). Still, current management practices rely heavily on insecticides targeting adult flies in crop fields (eg Van Timmeren and Isaacs 2013, Sial et al. 2019). However, *D. suzukii* can reinvade crop fields from non-crop refuge habitats (Klick et al. 2016, Tait et al. 2018, Tonina et al. 2018, Wang et al. 2019a), and areawide IPM strategies need to be developed for this highly mobile, polyphagous pest. Using parasitoids to suppress source populations of *D. suzukii* in non-crop habitats could be a key component of areawide control programs (Lee et al. 2019, Wang et al. 2020).

Parasitoid species such as *Asobara tabida* Nees (Braconidae), *Leptopilina heterotoma* (Thomson), and *L. boulardi* (Barbotin et al.) (Figitidae) are often found attacking drosophilid larvae in fermenting substrates (Fleury et al. 2009). However, these common larval parasitoids were rarely or never recovered from *D. suzukii* during surveys in the United States (Miller et al. 2015, Kamiyama et al. 2019, Huang et al. 2023, Neupane et al. 2024), Canada (Abram et al. 2020), Mexico (Cancino et al. 2015), Italy (Rossi Stacconi et al. 2015, Mazzetto et al. 2016), Spain (Gabarra et al. 2015), France (Kremmer et al. 2017), Switzerland (Knoll et al. 2017), and Brazil (Wollmann et al. 2016). Laboratory tests confirmed that they can parasitize *D. suzukii* but fail to develop because of the fly’s immune resistance (Chabert et al. 2012, Kacsoh and Schlenke 2012, Poyet et al. 2013). The only parasitoid species that consistently attack *D. suzukii* in its invaded range are the cosmopolitan pupal parasitoids *Trichopria drosophilae* Perkins (Diapriidae) and *Pachycrepoideus vindemiae* (Rondani) (Pteromalidae) (eg Rossi Stacconi et al. 2015, 2017, Wang et al. 2016a, b, Kaçar et al. 2017).

The near absence of larval parasitism in the invaded range prompted exploration for parasitoids in the native range in Asia. Three larval parasitoids caused > 70% parasitism in many locations: *Ganaspis kimorum* Buffington and *G. lupini* Buffington (formerly reported as *G.* nr. sp. *brasiliensis* (Ihering) [Hopper et al. 2024, Sosa-Calvo et al. 2024]), and *Leptopilina japonica* Novkovic & Kimura (Figitidae) (Daane et al. 2016, Girod et al. 2018a, Giorgini et al. 2019). Releases of *G. kimorum* began in 2021 in Italy (Lisi et al. 2022, Fellin et al. 2023) and the US after testing determined that it was specialized on *D. suzukii* (Stahl et al. 2024b) and *L. japonica* is now adventive in the United States and Canada (Abram et al. 2020, Beers et al. 2022, Gariepy et al. 2024). Concurrently, an adventive population of *G. kimorum* was also discovered first in British Columbia, Canada (Abram et al. 2020), and in neighboring Washington, USA (Beers et al. 2022). Crossing experiments showed reproductive compatibility between the released and the adventive population of *G. kimorum* (Stahl et al 2024a).

Naturally occurring parasitism of *D. suzukii* has not been thoroughly reported from California, where most fruit production in the US occurs. Here, we provide information on *D. suzukii* parasitism levels from multiple regions in California before releases of *G. kimorum* occurred or adventive populations of *L. japonica* were found, focusing on the effectiveness, diversity, and geographic distribution of the extant parasitoid fauna associated with *D. suzukii*. This information is critical to the implementation of sustainable management strategies or a classical biological control program. We also report on the preference of resident parasitoids for *D. suzukii* versus the closely related *D. melanogaster*.

## Materials and Methods

### Survey Locations

**Fig. 1.**
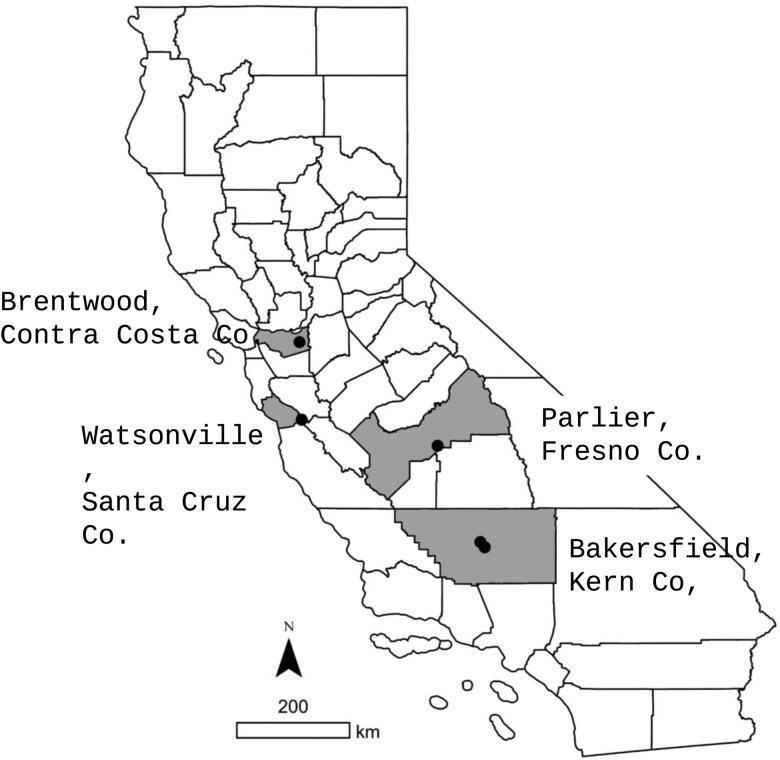
Location of the different sample sites within some of California’s major fruit growing regions.

Surveys using sentinel *D. suzukii* pupae and/or larvae, sentinel fruit, and/or fruit collections were conducted in California’s coastal and interior fruit production regions (Fig. 1, Table 1). The interior sites were in the upper (Brentwood, Contra Costa County), middle (Parlier, Fresno County), and lower (Bakersfield, Kern County) San Joaquin Valley, a major stone fruit production region where cherry has been severely impacted by *D. suzukii* (Haviland et al. 2016, Wang et al. 2019a). The coastal region was in Watsonville (Santa Cruz County), a key berry production region where *D. suzukii* was first detected in North America. The middle and lower San Joaquin Valley have hotter summers and colder winters than the upper Valley. In contrast, climatic conditions in the coastal region are mild throughout the year.

**Table 1.** Locations and survey methods for parasitoids of *D. suzukii* and other frugivorous Drosophilidae in California

Region	County	Site	Sample years	Survey method	Fruit	Bait^1^	Sample crops
Interior	Fresno	Parlier	2012–2014	Fruit samples	-	-	Various
Interior	Fresno	Parlier	2014	Sentinel traps	Banana	D.s. + D.m. pupae	Cherry, citrus, pomegranate
Interior	Fresno	Parlier	2017–2018	Sentinel traps	Banana	D.s. pupae	Cherry, citrus
Interior	Fresno	Parlier	2017–2018	Sentinel traps	Cherry, banana	Fruit only	Cherry, citrus
Interior	Contra Costa	Brentwood	2013	Sentinel traps	Banana	D.s. pupae	Cherry, peach, pear
Interior	Contra Costa	Brentwood	2013–2014	Fruit samples	-	-	Various
Interior	Kern	Bakersfield	2013	Sentinel traps	Artificial diet	D.s. larvae + pupae	Citrus
Coastal	Santa Cruz	Watsonville	2017	Sentinel traps	Raspberries	D.s. larvae + pupae	Blackberry, raspberry

^1^D.s. = *Drosophila suzukii*; D.m. = *Drosophila melanogaster*.

### Insect Colonies

Fly colonies were maintained to supply the sentinel larvae and pupae used in this study. Insect rearing and sample processing for Parlier and Brentwood locations occurred at the University of California’s Kearney Agricultural Research and Extension Center (KARE) in Parlier, CA. Laboratory colonies of both *D. suzukii* and *D. melanogaster* used for sentinel traps were established from 2013 field collections of infested or damaged cherries and peaches, respectively, and maintained under controlled conditions (22 ± 2 °C, 16L: 8D, 30–70% RH), with infusions of fresh field-collected material, during the 5-yr study. For Bakersfield surveys, insect rearing and sample processing were conducted at the University of California’s Bakersfield Extension Station, where a *D. suzukii* colony was established from sentinel blueberry fruit placed in citrus and cherry orchards near Edison, Kern County, and maintained under ambient laboratory conditions. Insect rearing and sample processing for the Watsonville surveys were conducted at the USDA-ARS facility in Albany, CA, where a *D. suzukii* colony (derived from the Parlier colony) was maintained under ambient room conditions (22 ± 2 °C, 14L: 10D, 30–60% RH). All fly colonies in these three locations were maintained on a standard artificial diet (described in Wang et al. 2016b).

### Sentinel Traps

Sentinel *D. suzukii* traps were used for the surveys in all locations and were constructed from the same materials unless otherwise noted. Traps were made of 16 oz (473 ml) clear plastic deli containers (11 × 11 cm), with ten 0.8 cm diameter holes on the side to allow flies and parasitoids to enter while preventing entry by large insects or other animals. To maintain moisture, the *D. suzukii* pupae were placed on a 3-cm-thick layer of agar medium (45 g Agar (Thermo Fisher Scientific, Inc., Waltham, MA) and 4.8 L distilled water) that contained no sugar or any nutritional compounds and was used purely to provide a substrate and to create a humid environment. Traps were covered by Pherocon 1C Trap lids (Trécé Inc., Adair, OK) to protect the bait from rain or direct sunlight and were hung by a metal wire from tree branches or trellis wires 1 to 1.5 m above the ground. A ring of Tanglefoot (The Scotts Company LLC, Marysville, OH) (Parlier, Brentwood) or petroleum jelly (Watsonville) was placed around the metal wire as a barrier for foraging ants. Fruit used in traps, methods for inoculating fruit with sentinel *D. suzukii,* and duration of placement in the field varied between sample dates and regions (Table 1), as described below.

### Sample Processing

For Parlier and Brentwood surveys, collected fruit and fruit from sentinel traps were taken to the laboratory and placed in plastic ventilated containers for the emergence of fly pupae. Fruit was placed on a wire platform over a piece of tissue or filter paper to absorb any liquid that accumulated. Collected fruits were typically placed in containers in groups of 1–4 for large fruit (eg citrus, peach), 5–15 for medium fruit (eg cherries, raspberries) or 16–30 for small fruit (eg blueberries). A sub-sample of 5–10 fruit were weighed for each fruit species to standardize fly density per 5 g fruit (roughly the size of an average cherry fruit) across different fruit species. Emerged pupae were collected every 2–3 d. Only those that pupated within 2 wk following field collections were collected to exclude second-generation flies. For the Watsonville survey, fruit from sentinel traps were dissected within 3 d of collection to collect drosophilid pupae.

Collected pupae of *D. suzukii* and other Drosophilidae (if present) were separated and placed on pieces of filter paper in petri dishes (5 cm diameter × 1 cm high or 10 cm × 2 cm high) until the emergence of flies and parasitoids. After all parasitoids and flies had emerged, all dead pupae were dissected. They were first reconstituted in water for 1–2 d before they were dissected under a microscope to determine the presence or absence of recognizable fly or parasitoid cadavers (pharate adults or larvae). Emerged parasitoids were preserved in 95% alcohol for identification. A sub-sample of each common parasitoid species was sent to and confirmed by Dr. Matthew Buffington (USDA Agricultural Research Service, Systematic Entomology Laboratory, Washington, DC, USA). Voucher specimens were deposited in the National Insect Collection, USA.

### Parlier Survey

Fruit sampling was conducted from 2012 to 2014 in Parlier at the KARE research farm on 11 common crops (in temporal sequence of fruit ripening, see Wang et al. 2019a) including cherry, peach, nectarine, plum, pear, grape, fig, pomegranate, apple, persimmon, and orange (Table 2). Only obviously infested or damaged fruits (with open wounds or cracks, or split) were collected as available, as the fruits entered susceptible ripening stages for *D. suzukii* oviposition. The numbers of fruit collected varied, depending on fruit size and availability (Table 2).

**Table 2.** Numbers of *D. suzukii* (Ds) and other Drosophilidae (Non-Ds) recovered and percentage parasitism (Par %) by *P. vindemiae* (Pv), *T. drosophilae* (Td) and *L*. *boulardi* (Lb) from fruit samples in two different locations in California

Location	Year	Month	Fruit species	Fruits collected	*Ds* per 5g fruit	Ds Par (%)		Non-Ds per 5g fruit	Non-Ds Par (%)		
						Pv	Td		Pv	Td	Lb
Brentwood	2013	May	Cherry	18	0.111	0	0	0			
Brentwood	2013	May	Loquat	25	0.100	0	0	2.100	0	0	2.86
Brentwood	2013	May	Nectarine	5	0.010	0	0	0			
Brentwood	2013	May	Peach	5	0			0.200	0	0	0
Brentwood	2013	Sep	Pear	15	0			0.243	0	0	13.70
Brentwood	2014	Jul-Sep	Peach	15	0			0.200	0	0	0
Brentwood	2014	Jul-Sep	Pear	10	0.530	0	0	0.495	7.07	29.29	0
Brentwood	2014	Sep	Cactus	20	0.013	0	0	0			
Parlier	2012	May-Jun	Cherry	793	0.941	0	0	0.184	0	0	19.86
Parlier	2012	Jun	Nectarine	10	0			2.500	0	0	0
Parlier	2012	Jul-Aug	Peach	25	0.018	0	0	3.274	0	0	6.72
Parlier	2012	Aug	Fig	20	0.083	0	0	2.583	0	0	0
Parlier	2013	Mar	Orange	15	0			0.520	0	0	0
Parlier	2013	May	Cherry	353	0.031	0	0	0			
Parlier	2013	Jun-Sep	Peach	30	0.003	0	0	0.170	0	0	0
Parlier	2013	Sep-Dec	Apple	65	0.002	0	0	0.314	1.96	0.49	0
Parlier	2013	Sep-Dec	Plum	60	0.004	0	0	0.233	5.02	0	0
Parlier	2013	Sep-Nov	Fig	30	1.222	0	0	0.211	0	0	5.26
Parlier	2013	Sep-Oct	Grape	1000	0			0.075	0	0	0
Parlier	2013	Nov-Dec	Pomegranate	20	0.030	0	0	0.625	1.60	0	0
Parlier	2014	Jan	Fig	5	7.333	0	0	9.400	7.09	21.99	0
Parlier	2014	Jan-Apr	Pomegranate	70	0			2.076	20.23	1.31	0.03
Parlier	2014	Jan-Mar	Apple	70	0.001	0	0	0.485	0.88	0.29	0
Parlier	2014	Mar	Orange	60	0			1.563	4.00	14.99	0
Parlier	2014	May-Jun	Cherry	909	0.113	7.77	1.94	0.067	0	1.64	0
Parlier	2014	Dec	Persimmon	35	0.003	0	0	0.886	0	0	0

Baited sentinel traps were deployed in Parlier in the spring of 2014 and again in the winter and spring of 2017–2018 at the research farm of KARE. A 20 g banana slice was placed inside each container to provide fruit volatiles that may be needed to attract parasitoids. To assess host species preference by pupal parasitoids, sentinel traps baited with pupae of both *D. suzukii,* and the common host *D. melanogaster* were deployed every 6–22 d on 6 sample dates from 19 March to 23 May 2014 in cherry, citrus, and pomegranate orchards. Each trap contained 20 *D. suzukii* pupae and 20 *D. melanogaster* pupae. A total of 120 traps were deployed, including 30 in cherry orchards, 30 in pomegranate orchards, and 60 in citrus orchards (ie 5 or 10 traps per sample date per orchard). Traps (each 5 trees apart) were hung on trees and were left in the field for 7–10 d. After collection, pupae of host species (*D. suzukii*, *D. melanogaster*) were removed and held separately for emergence as described above. Traps baited with 20 newly formed *D. suzukii* pupae were deployed every 6–34 d from 9 December 2017 to 30 May 2018. On each date, five or ten traps (each 5 trees apart) were hung on cherry trees during the spring or citrus trees during the other seasons and remained in the field for 7–10 d.

From 2017 to 2018, traps baited with uninfested fruit only were used at the research farm at KARE to attract frugivorous drosophilids and associated parasitoids. Traps were baited with 5 cherries (approximately 20 g). and placed in cherry orchards during the cherry ripening seasons (May and June) or baited with 20 g banana slices and placed in citrus orchards for the remaining sample dates (bananas were used because they were readily available year-round). Traps were deployed and collected weekly (from spring through early fall) or biweekly (for the remaining seasons) from 10 May 2017 to 6 April 2018. On each sample date, 10 traps were deployed in trees that were each 5 trees apart.

### Brentwood Survey

In Brentwood, surveys using baited sentinel traps were conducted in the spring and fall of 2013. Each trap contained 20 newly formed *D. suzukii* pupae and a 20 g banana slice to provide fruit volatiles. Five traps were placed along the edge (< 10 m) of riparian areas and ~10 m apart directly adjacent to various commercial fruit orchards (cherry, pear, and peach). Potential host plants including wild *Rubus*, Klamath plum (*Prunus subcordata* Benth.), cherry plum (*P. cerasifera* Ehrh.), and prickly pear cactus *Opuntia* sp. were present in the riparian area.

Fruit sampling was conducted from 2013 to 2014 near Brentwood on four common crops, including cherry, nectarine, peach, and pear, and on two non-crop fruits (loquat and cactus) in riparian areas surrounding stone fruit orchards (Table 2). Only obviously infested or damaged fruits were collected. The numbers of fruit collected varied, depending on fruit size and availability (Table 2).

### Bakersfield Survey

In Bakersfield, baited sentinel traps (without fruit) were deployed in 2013 in two conventionally managed navel orange orchards (referred to as Edison and Beale) during winter from 6 February to 20 March, spring from 1 to 21 May, and fall from 2 October to 27 November. To prepare each trap, approximately 200 ml of artificial diet was placed in the bottom of a 591 ml square plastic container with a Snap-On lid, and 40 mixed-gender adult *D. suzukii* were added to each container for 12 d to inoculate the diet with mixed-stage *D. suzukii* larvae and newly formed pupae. The lid was then removed, and traps were placed in the field. Five traps were placed underneath randomly selected citrus trees at each orchard on each sample date. Traps were field-deployed for 7 d, recovered, and stored with the lid replaced under ambient laboratory conditions. After 5 wk, parasitoids were collected and identified. This period of time was found to be sufficient for all parasitoids to emerge, but not sufficient for a second generation of parasitism to occur.

### Watsonville Survey

In Watsonville, traps were deployed in 2017 in nine commercial organic cane berry (blackberry, raspberry) fields. Each site was sampled once during each of three sample periods: 28 July to 14 August; 21 September to 25 October; and 8 December. Traps contained 20 g of organic raspberries, which were inoculated by exposing them to *D. suzukii* adults in the Albany laboratory for 48 h before deploying the traps in the field. On sample dates from July to October, traps were placed at 3–6 distances along transects at 30 m intervals, starting 10 m from the edge of adjacent riparian habitat and extending 70 to 160 m into the berry fields, depending on the size of sites. Three traps were placed approximately 20 m apart at each transect distance (9–18 traps total per site). In December, muddy conditions limited the accessibility of sites, and three traps were placed at one transect distance at each site, 100 m into berry fields (three traps per site). Traps were left in the field for 7–10 d.

### Data Analysis

Samples that yielded neither *D. suzukii* nor other drosophilid species were excluded from the analyses. Data were analyzed in R version 4.2.3 (R Development Core Team 2023) . For fruit collections in Parlier from 2012 to 2014, counts of total  *D. suzukii* and other Drosophilidae were pooled for each fruit species on each sample date (numbers were low), and mean density per 5 g fruit was calculated. Parasitism rates were then analyzed using generalized linear models (GLM) with negative binomial errors and the glm.nb function in R to account for overdispersion, including host type (*D. suzukii*, other Drosophilidae) and host density as explanatory factors and a host type × host density interaction term. All other data were analyzed using repeated measures generalized linear mixed models (GLMM), with sample date/period as an explanatory factor, using the glmer function in R with either Poisson errors (for parasitoid numbers) or binomial errors (for proportion parasitism), or using the glmer.nb function with negative binomial errors when overdispersion was detected. For traps baited with 20 *D. suzukii* pupae that were deployed in Parlier in spring 2014, data from the three orchards (citrus, cherry, pomegranate) were analyzed separately, and host species (*D. suzukii*, *D. melanogaster*) was included as an additional explanatory factor. For the Bakersfield survey, data were analyzed separately for each site (Edison, Beale). For the Watsonville survey, additional explanatory variables included host density and host type (*D. suzukii*, other Drosophilidae). Trap data were transformed to insects per trap per week, as needed. Finally, all recovered parasitoids in each location were pooled to show the regional distribution and dominance of these parasitoids.

## Results

### Parlier Survey

In fruit sampling that was conducted in Parlier from 2012 to 2014, *D. suzukii* was recovered consistently from cherries but its density on other sampled fruit was very low (< 0.01 per 5 g fruit) except on a few samples of damaged figs and pears (Table 2). The density of other drosophilids also varied among sampled fruits (ranging from 0.0 to 9.4 per 5 g fruit). In Parlier, parasitism on *D. suzukii* was recorded on only one sample date, on cherry from May to June, when both *T. drosophilae* and *P. vindemiae* were collected (Table 2). For other drosophilids, parasitism by both pupal parasitoids was recorded on eight fruit types on multiple sample dates, and *L. boulardi* was collected on fig in September to December and pomegranate in January to April (Table 2). In Brentwood, no parasitism was recorded on *D. suzukii* on any sample dates, but both pupal parasitoids were collected from other drosophilids on pear from July to September, and *L. boulardi* was collected on fig from September to December and on pomegranate from January to April (Table 2). Mean parasitism was affected by host type and host density for both *P. vindemiae* (host type: χ^2^ = 127.17, df = 2, *P* < 0.001; host density: χ^2^ = 12.66, df = 1, *P* < 0.001; host type × host density interaction: χ^2^ = 31.18, df = 1, *P* < 0.001) and *T. drosophilae* (host type: χ^2^ = 80.72, df = 1, *P* < 0.001; host density: χ^2^ = 18.41, df = 1, *P* < 0.001; host type × host density interaction: χ^2^ = 8.16, df = 1, *P* = 0.004). Parasitism levels for both of these parasitoid species were higher on other drosophilid species (Table 2). Parasitism levels for *L. boulardi* were too low for analysis.

**Fig. 2.**
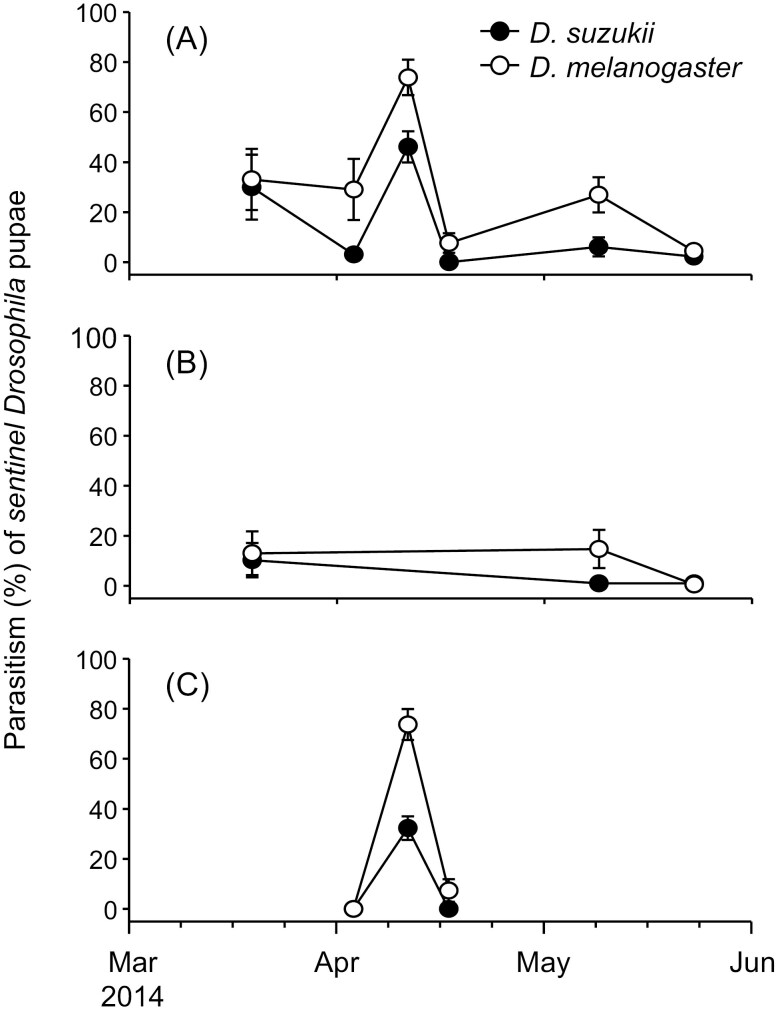
Parasitism of *P. vindemiae* on *D. suzukii* and *D. melanogaster* over time in (A) citrus (B) cherry or (C) pomegranate orchards in Parlier, California in the spring of 2014. Data are means ± SE.

In traps baited with both *D. suzukii* and *D. melanogaster* pupae that were deployed in Parlier in spring 2014, collected parasitoids included 771 *P. vindemiae* (98.3% of collected parasitoids) and 13 *T. drosophilae*. Parasitism levels of *T. drosophilae* were too low for analysis. Proportion parasitism by *P. vindemiae* was affected by host species in all three orchards (citrus: χ^2^ = 65.31, df = 1, *P* < 0.001; cherry: χ^2^ = 10.87, df = 1, *P* < 0.001; pomegranate: χ^2^ = 45.88, df = 1, *P* < 0.001), sample date in all three orchards (citrus: χ^2^ = 110.69, df = 1, *P* < 0.001; cherry:χ^2^ = 26.67, df = 1, *P* < 0.001; pomegranate: χ^2^ = 7.21, df = 1, *P* = 0.007), and the host species × sample date interaction in citrus and cherry but not in pomegranate (citrus: χ^2^ = 3.94, df = 1, *P* = 0.047; cherry:χ^2^ = 5.17, df = 1, *P* = 0.02; pomegranate: χ^2^ = 0.49, df = 1, *P* = 0.49) (Fig. 2). Parasitism levels were highest in April in citrus and pomegranate and were higher on *D. suzukii* than *D. melanogaster* on most sample dates at all three orchards (Fig. 2).

**Fig. 3.**
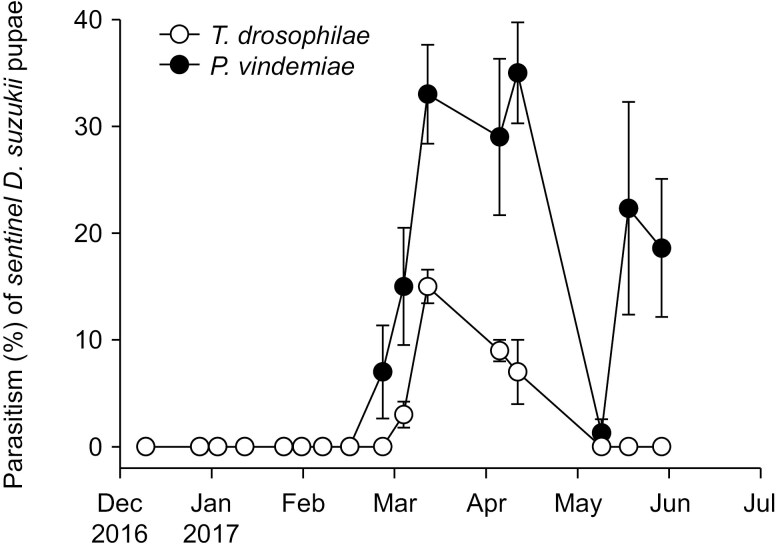
Parasitism of *D. suzukii* by *P. vindemiae* and *T. drosophilae* in the winter and spring of 2016-2017 in Parlier, California. Data are means ± SE.

In traps baited with 20 *D. suzukii* pupae from winter and spring of 2016-2017, a total of 296 *P. vindemiae* (89.7% of collected parasitoids) and only 34 *T. drosophilae* were collected. Parasitism levels of *T. drosophilae* were too low for analysis. Parasitism by *P. vindemiae* changed over time (χ^2^ = 134.04, df = 1, *P* < 0.001), and occurred from February to May, but not in December and January, and reached a maximum of 35.0% on 6 April (Fig. 3).

**Fig. 4.**
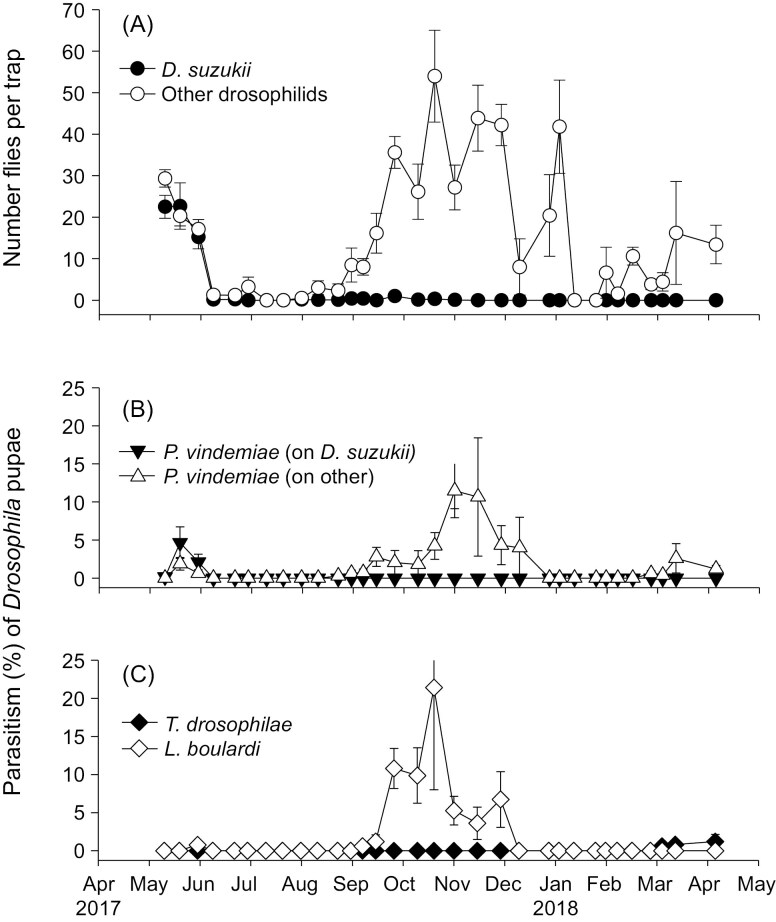
(A) Number of *D. suzukii* (Ds) and other Drosophilidae (Non-Ds) per sentinel fruit trap, (B) parasitism by *P. vindemiae* (Pv) on Ds and Non-Ds, and (C) parasitism of *T. drosophilae* (Td) and *L*. *boulardi* (Lb) on Non-Ds from 2017 to 2018 in Parlier, California. Cherry was used as bait during cherry ripening and traps were placed in cherry orchards, while banana was used as bait in traps placed in citrus orchards for the rest of the season. Data are means ± SE.

In traps baited with fruit only that were deployed in Parlier from 2017 to 2018, *D. suzukii* was collected almost exclusively in May in cherries, when cherry was used as bait, and was absent during the other months in citrus orchards when banana was used as bait, while numbers of other Drosophilidae had two peaks in the spring and fall (Fig. 4A). Only *P. vindemiae* was recovered from *D. suzukii* with parasitism ranging from 0 to 4.6%, and levels were too low for analysis (a total of 79 were collected) (Fig. 4B). Parasitoids collected from other Drosophilidae included 862 *P. vindemiae* (57.4%), 13 *T. drosophilae* (0.9%) and 628 *L. boulardi* (41.8%). Proportion parasitism by *P. vindemiae* and *L. boulardi* on other drosophilids changed over time (*P. vindemiae*: χ^2^ = 35.75, df = 1, *P* < 0.001; *L. boulardi*: χ^2^ = 8.77, df = 1, *P* = 0.003), while parasitism levels by *T. drosophilae* on other drosophilids were too low for analysis (Fig. 4C). All three parasitoids were more active during fall and spring.

### Brentwood Survey

**Fig. 5.**
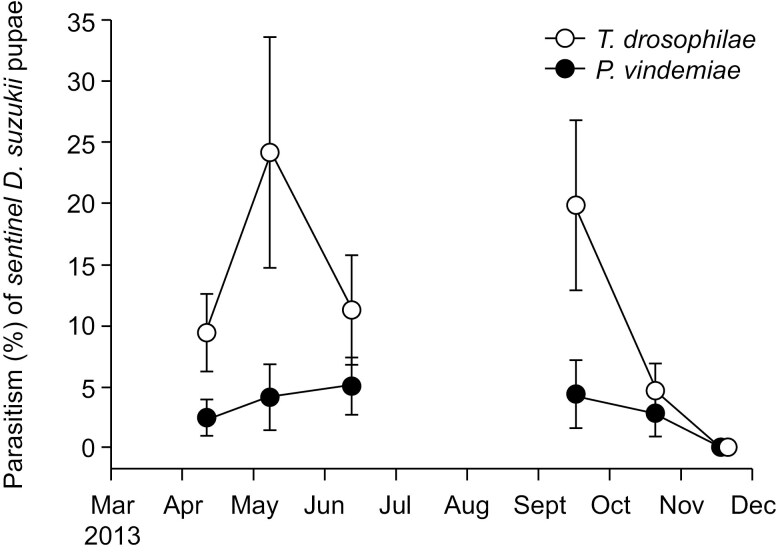
Parasitism of *D. suzukii* by *P. vindemiae* and *T. drosophilae* in sentinel traps in the spring and fall of 2013 in Brentwood, California. Data are means ± SE.

In traps baited with 20 *D. suzukii* pupae that were deployed in Brentwood in 2013, a total of 64 *T. drosophilae* (78.0%) and 18 *P. vindemiae* were recovered. Proportion parasitism by *T. drosophilae* was affected by sample date (χ^2^ = 35.75, df = 1, *P* < 0.001), while parasitism levels by *P. vindemiae* were too low for analysis. The numbers of both parasitoids increased in mid-spring and fall and then declined after early summer and later fall (Fig. 5).

### Bakersfield survey

**Fig. 6.**
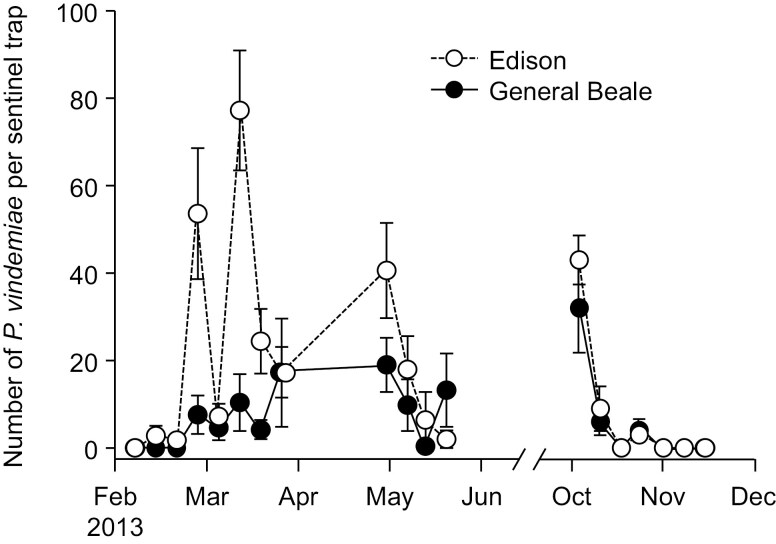
Number of *P. vindemiae* collected per sentinel *D. suzukii* trap in (A) spring and (B) fall of 2013 in Bakersfield, California. Data are means ± SE.

In traps baited with mixed-stage *D. suzukii* that were deployed in Bakersfield in 2013, *P. vindemiae* was the only parasitoid recovered from *D. suzukii* (Fig. 5); a total of 1256 and 415 were collected in spring, and 273 and 210 were collected in fall at the Edison and Beale sites, respectively. Numbers of *P. vindemiae* changed over time at both sites in spring (Edison: χ^2^ = 39.59, df = 1, *P* < 0.001; Beale: χ^2^ = 92.75, df = 1, *P* < 0.001) and fall (Edison: χ^2^ = 40.67, df = 1, *P* < 0.001; Beale: χ^2^ = 22.97, df = 1, *P* < 0.001). Seasonal trends were similar in both monitored citrus orchards; numbers increased in mid-March and then decreased after May and then increased in the fall before decreasing after October (Fig. 6).

### Watsonville Survey

**Fig. 7.**
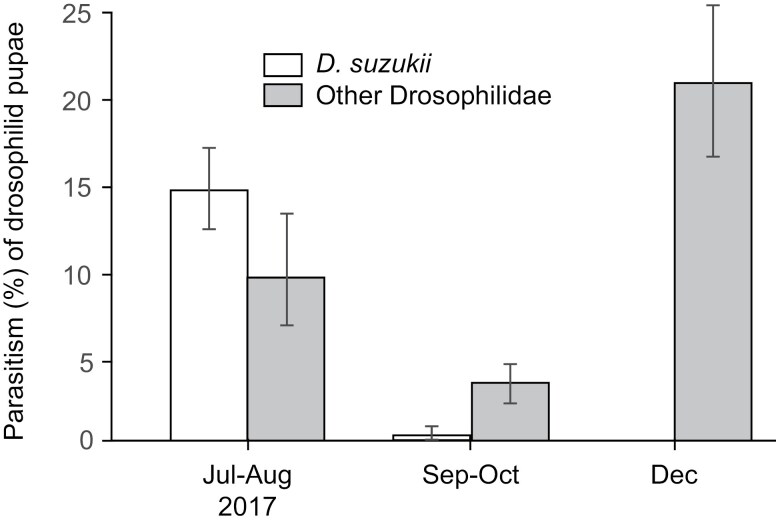
Parasitism by *T. drosophilae* on *D. suzukii* and other Drosophilidae during the summer and fall of 2017 in Watsonville, California. Data are means ± SE.

In traps inoculated with immature *D. suzukii* that were placed in Watsonville, a total of 895 *T. drosophilae* and 38 *P. vindemiae* were recovered from *D. suzukii*, and 89 *T. drosophilae*, 2 *P. vindemiae* and 31 *L. heterotoma* were collected from other drosophilids. Parasitism rates by *P. vindemiae* and *L. heterotoma* were too low for analysis. Parasitism by *T. drosophilae* was affected by sample period (χ^2^ = 95.26, df = 5, *P* < 0.001), host density (χ^2^ = 30.14, df = 5, *P* < 0.001), host species (χ^2^ = 15.18, df = 3, *P* = 0.002), and interactions between sample period and host density (χ^2^ = 18.00, df = 3, *P* < 0.001) and sample period and host species (χ^2^ = 15.33, df = 3, *P* = 0.002), but not by the host density × host species interaction (χ^2^ = 3.80, df = 2, *P* = 0.15) or the sample date × host density × host species interaction (χ^2^ = 1.27, df = 2, *P* = 0.53). Parasitism by *T. drosophilae* was consistently higher on *D. suzukii* than other Drosophilidae and was highest on *D. suzukii* in December and on other drosophilids from July to August (Fig. 7).

### Regional Patterns

**Fig. 8.**
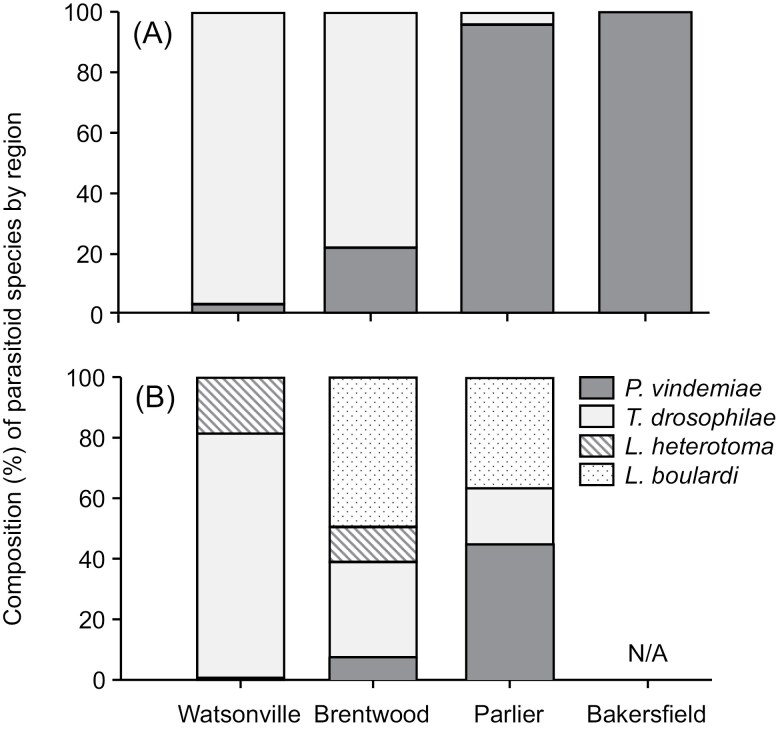
Regional dominance (percentage) of *P. vindemiae* and *T. drosophilae* collected from (A) *D. suzukii* and (B) other Drosophilidae and *L. heterotoma* and *L*. *boulardi* collected from (C) other Drosophilidae in Watsonville, Brentwood, Parlier and Bakersfield, California. Data were pooled from different collections in each location.

*Pachycrepoideus vindemiae* dominated in the interior valley and its dominance increased further south while *T. drosophilae* dominated in the coastal region (Fig. 8). *Leptopilina boulardi* was present only in Parlier while *L*. *heterotoma* was present only in Watsonville but both species overlapped in Brentwood (Fig. 8).

## Discussion

Our surveys revealed two larval (*L. boulardi* and *L. heterotoma*) and two pupal (*T. drosophilae* and *P. vindemiae*) drosophilid parasitoids in California, and absence of adventive *L. japonica* or other Asian larval parasitoids. The pupal parasitoids were found on *D. suzukii* and other related drosophilids whereas the larval parasitoids were not found on *D. suzukii* and only on other Drosophilidae. The seasonal occurrence of these four common parasitoids was similar, with two peaks in activity in spring and fall. However, they showed distinctly different regional dominance patterns in California.

We found *L. boulardi* was the dominant species throughout the San Joaquin Valley (Parlier, Bakersfield), while *L. heterotoma* was the dominant species in the coastal region (Watsonville), and both species overlapped in the upper San Joaquin Valley (Brentwood). The geographic distributions of these two parasitoids have been shown to differ in other regions. For example, in Southern France *L. boulardi* was more abundant in the south while *L. heterotoma* was more abundant in the north, with their distributions overlapping in the middle (Fleury et al. 2009). In Tunisia, both species were present at different times of the season (Carton et al. 1991). The stark difference we uncovered between the interior and coastal regions in the relative abundances of these two parasitoids indicates that different thermal tolerances may play a role in determining the regional patterns of these two parasitoids in California. *Leptopilina boulardi* enters diapause as a prepupa (Hertlein 1986), while *L. heterotoma* overwinters as a quiescent adult (Carton et al. 1991), suggesting that they are adapted to different climatic conditions.

*Leptopilina boulardi* is a specialist of frugivorous *Drosophila* while *L. heterotoma* is one of the most generalist larval *Drosophila* parasitoids (Fleury et al. 2009). However, neither species appears capable of developing successfully on *D. suzukii* in laboratory tests (Chabert et al. 2012, Kacsoh and Schlenke 2012, Poyet et al. 2013, Mazzetto et al. 2016), although one study reported partial success of a northern Italian *L. heterotoma* population in developing on *D. suzukii* in artificial diet (Rossi Stacconi et al. 2015). Also, studies have reported occasional recoveries of *L. boulardi* from *D. suzukii* in Brazil and Argentina (eg Wollmann et al. 2016, Buonocore-Biancheri 2024). In California, both larval parasitoids were caught in high numbers in apple cider vinegar traps that were used to monitor *D. suzukii*, and a portion of captured flies (< 5%) had black capsules inside their abdomens, suggesting immune responses occurred in *D. suzukii* against these larval parasitoids (Wang et al. 2016c). The parasitoid’s virulence and/or the *D. suzukii*’s resistance may vary among regions. It is possible that populations of larval parasitoids in some regions will adapt to *D. suzukii* eventually.

We showed that the pupal parasitoid *T. drosophilae* was dominant in the coastal region but was almost absent in the lower San Joaquin Valley, where *P. vindemiae* was the only species found on *D. suzukii*. Both *P. vindemiae* and *T. drosophilae* are cosmopolitan, widely reported in association with *D. suzukii* and various other drosophilids worldwide (Mitsui et al. 2007, Mitsui and Kimura 2010, Chabert et al. 2012, Gabarra et al. 2015, Miller et al. 2015, Rossi Stacconi et al. 2015, Daane et al. 2016, Mazzetto et al. 2016, Knoll et al. 2017, Zhu et al. 2017, Giorgini et al. 2019, Abram et al. 2020). However, *P. vindemiae* has been reported more widely than *T. drosophilae*. For example, in Europe, *P. vindemiae* was present in all surveyed regions, whereas *T. drosophilae* was collected at only 1 of the 8 sampled locations in Switzerland (Knoll et al. 2017). Both species were present in Italy (Rossi Stacconi et al. 2017, Gugliuzzo et al. 2024). Similarly, in North America, only *P. vindemiae* was found in British Columbia, Canada (Abram et al. 2020), and in Oregon (Miller et al. 2015) and Michigan (Huang et al. 2023) in the United States, but both species were found in Georgia in the United States (Neupane et al. 2024) and Mexico (Cancino et al. 2015). Wang et al. (2018b) compared the thermal tolerances of these two species and showed that *P. vindemiae* could tolerate a wider temperature range than *T. drosophilae*. Under favorable temperatures for both species, *T. drosophilae* seems to be more effective and competitive than *P. vindemiae* (Wang et al. 2016b, Kaçar et al. 2017, Rossi Stacconi et al. 2017). These results seem to align with the findings from the current study and from earlier surveys.

Both *P. vindemiae* and *T. drosophilae* are generalist parasitoids; the former can attack various cyclorrhaphous dipteran species including tephritids (Wang and Messing 2004a), whereas the host range of the latter appears limited to Drosophilidae (Carton et al. 1976, Wang et al. 2021). We showed that *P. vindemiae* preferred to attack *D*. *suzukii* over *D. melanogaster* when both host species were included in sentinel traps. This preference may be related to the larger size of *D. suzukii* relative to *D. melanogaster*. The body size of emerging adult females of both *P. vindemiae* and *T. drosophilae* increased with host size (Wang and Messing 2004b, Wang et al. 2016a, b, 2021, Woltering et al. 2019), and in laboratory tests, *T. drosophilae* preferred to attack larger over smaller hosts, and more offspring successfully developed from the larger *D. suzukii* than the smaller *D. melanogaster* (Wang et al. 2016a, b, Woltering et al. 2019). However, *P. vindemiae* did not prefer *D*. *suzukii* over *D. melanogaster* when they pupated naturally inside fruit or soil (Wang et al. 2016b), suggesting that it is unable to precisely estimate the size of hosts that are not fully exposed. Also, there may be trade-offs between offspring fitness and other factors such as host handling time. For example, it took much longer for *P. vindemiae* to drill through a larger tephritid puparium than a smaller *Drosophila* puparium, and this difference seemed to offset any benefits conferred by the larger host (Wang and Messing 2004b).

The co-occurrence of *D. suzukii* with other drosophilids in collected fruits in the current study could have implications for biological control of *D. suzukii*. *Drosophila melanogaster* and *D. simulans* were the most common species on damaged fruits (ca. > 90%). Although *D. suzukii* likely exploits fruit resources in the ripe or ripening stages before they are available to other drosophilids such as *D. melanogaster*, it may compete with other Drosophilidae in some circumstances (Wang et al. 2016c, Kremmer et al. 2017). When forced to share resources in the laboratory, *D. melanogaster* outcompetes *D. suzukii* (Dancau et al. 2017). This may have important implications for biological control of *D. suzukii*. Most larval drosophila parasitoids can attack and develop from *D. melanogaster*, including the released *G. kimorum* and adventively established *L. japonica* in the United States (Biondi et al. 2021, Daane et al. 2021). Using *D. melanogaster* as an alternate host may help introduced larval parasitoids persist, especially during the post-fruit seasons when *D. suzukii* is in low numbers, eventually leading to an increased impact on *D. suzukii* populations. It is also possible that competition between introduced and resident larval parasitoids on *D. melanogaster* may drive introduced larval parasitoids to *D. suzukii* as ‘competition-free’ space. Also, *P. vindemiae* was able to successfully develop on *D. suzukii* pupae containing all preimaginal stages of *G. kimorum*, while *T. drosophilae* was able to successfully develop on *D. suzukii* puparia containing early instars of *G. kimorum* (Hougardy et al. 2022, Lisi et al. in press). These pupal parasitoids have the potential to negatively affect the larval parasitoids, although more research is required to assess these interesting possibilities.

There were a number of flaws in the project that must be addressed. Foremost was the evolving, and therefore inconsistent, sampling methods used. When the surveys were initiated, standardized sampling for *D. suzukii* parasitoids had not been addressed (see Abram et al. 2022). We used three different methods for sampling parasitoids, and some appear to have been more effective than others, leading to changes in methodology over the course of our collections. Our surveys suggest that banana bait is not very attractive to *D. suzukii*, although this method has been extensively used in surveys of *Drosophila* parasitoids (eg Mitsui et al. 2007, Mazzetto et al. 2016, Knoll et al. 2017). While this method is appropriate for surveys of generalist drosophilids and their parasitoids (Daane et al. 2016, Girod et al. 2018a, Giorgini et al. 2019), other fruits such as cherry seem to be a better bait for *D. suzukii* parasitoids. Another issue was the seasonal period of sample collections, which varied largely because of crop harvest periods and, therefore, the predicted presence of *D. suzukii* and its parasitoids. Still, this results in early-season sampling at sites with cherries, mid-season sampling with berries, and late-season sampling with citrus.

In conclusion, our California surveys showed that naturally occurring parasitism of *D. suzukii* was low and there is an absence of resident parasitoids attacking *D. suzukii* larvae. These results are consistent with surveys in other regions of the fly’s invaded range (e.g., Miller et al. 2015, Rossi Stacconi et al. 2015, Kamiyama et al. 2019). Extant parasitoids (mainly pupal parasitoids) seem to be insufficient to provide natural suppression of *D. suzukii* or through augmentation release (eg Hogg et al. 2022). In contrast, foreign explorations for native parasitoids in China, Japan and South Korea have discovered a diverse suite of larval parasitoids associated with *D. suzukii* (Daane et al. 2016, Girod et al. 2018a, Giorgini et al. 2019), with *Asobara japonica* Belokobylskij (Hymenoptera: Braconidae), *G. kimorum* and *L. japonica* being the most common. The biology and behavior of these Asian larval parasitoids have recently been evaluated in quarantine (Biondi et al. 2017, Girod et al. 2018b, Wang et al. 2018a, b, 2019b, Hougardy et al. 2019, Daane et al. 2021). Future direction of biological control of *D. suzukii* may focus on the release and establishment of the host-specific *G. kimorum* and the use of *L. japonica* (Stahl et al. 2024b).
